# A Study of Wideband Energy Reflectance in Patients with Otosclerosis: Data from a Chinese Population

**DOI:** 10.1155/2019/2070548

**Published:** 2019-08-14

**Authors:** Suju Wang, Wenyang Hao, Chunxiao Xu, Daofeng Ni, Zhiqiang Gao, Yingying Shang

**Affiliations:** Department of Otorhinolaryngology, Peking Union Medical College Hospital, Chinese Academy of Medical Sciences and Peking Union Medical College, Beijing 100730, China

## Abstract

**Objective(s):**

The purpose of this study was to explore the effectiveness of wideband acoustic immittance (WAI) in the diagnosis of otosclerosis by comparing the differences in the energy reflectance (ER) of WAI between patients with otosclerosis and age- and gender-matched normal hearing controls in the Chinese population.

**Methods:**

Twenty surgically confirmed otosclerotic ears were included in the otosclerotic group. The ER of WAI at ambient and peak pressures, resonance frequency, and 226-Hz tympanogram were collected prior to surgery using a Titan hearing test platform (Interacoustics A/S, Middelfart, Denmark). All diagnoses of otosclerosis in the tested ear were confirmed by surgery after the measurements. Thirteen normal adults (26 ears) who were age- and gender-matched with the otosclerotic patients were included as the control group.

**Results:**

At peak pressure, the ERs of otosclerotic patients were higher than those of the control group for frequencies less than 4,000Hz and were lower for frequencies greater than 4,000Hz. In addition, within the analyzed frequencies, the differences observed at 2,520Hz was statistically significant (*p<0.05/16=0.003, Bonferroni corrected*). At ambient pressure, the differences observed at 1,260 and 6,350Hz were statistically significant (*p<0.05/16=0.003, Bonferroni corrected*). Although the differences between the otosclerotic and control groups exhibited similar trends to those in studies implemented in Caucasian populations, the norms in the present study in the control group were different from those in the Caucasian populations, suggesting racial differences in WAI test results. Regarding the middle ear resonance frequency, no significant difference was observed between the two groups (*P>*0.05).

**Conclusion:**

WAI can provide valuable information for the diagnosis of otosclerosis in the Chinese population. Norms and diagnostic criteria corresponding to the patient's racial group are necessary to improve the efficiency of WAI in the diagnosis of otosclerosis.

## 1. Introduction

Otosclerosis is a lesion occurring in the middle ear and bony labyrinth, and its main clinical manifestations are progressive conductive or mixed hearing losses with a normal eardrum. The different stages of pathological alterations in the stapes footplate, including localized fixation of the anterior part of the footplate to bony ankylosis of the entire circumference of the annular ligament, lead to different levels of conductive hearing loss [1]. As one of the most effective tools for evaluating middle ear function, acoustic immittance measurements have been used to investigate the mechanical characteristics of the middle ears in otosclerotic patients. Traditional single-frequency and multifrequency tympanometry have revealed that the immittance characteristics in typical cases of otosclerosis include an increase in low-frequency acoustic stiffness and a decrease in static immittance amplitude [2, 3]. However, Muchink et al. [4] employed 220- and 660-Hz probe tones for measurements in otosclerotic patients, and only one-third of otosclerotic patients had immittance values below the normal range, while all other patients showed immittance values within or beyond the normal range. These results indicate that there is a large overlap between the static immittance of otosclerotic and normal ears, which suggests that the effectiveness of the traditional tympanogram (226Hz) in the diagnosis of otosclerosis is low [3–5]. Therefore, a middle ear test method with higher sensitivity is needed to detect subtle changes in sound conductance characteristics in patients with ossicular-chain disorders.

The gold standard for the diagnosis of conductive hearing loss induced by otosclerosis is the discovery of stapes footplate fixation during surgery. However, other conductive hearing losses (e.g., ossicular disarticulation and superior canal dehiscence) could also display similar characteristics in pure tone audiometry (PTA) and tympanograms obtained with a conventional 226-Hz probe tone [6–8]. Among the causes of conductive hearing losses, some require entirely different therapy. For instance, superior canal dehiscence indicates a dehiscence of the bone overlying the superior canal, which can result in apparent air-bone gap and requires resurfacing or plugging of the affected superior canal instead of a middle ear surgery [7]. Thus, an accurate diagnosis of otosclerosis prior to surgery is critical to avoid unnecessary procedures. Typical computed tomography (CT) has low sensitivity in the diagnosis of otosclerosis. High-resolution CT has increased sensitivity (74%); however, in the inactive form phase, otosclerotic lesions have the same density as the surrounding bone, which could lead to false-negative images, in addition to the radiation exposure and higher cost associated with CT [9]. Therefore, a low-cost and noninvasive method for differentiating otosclerosis from other conductive hearing losses would be beneficial in clinical practice.

Wideband acoustic immittance (WAI) can measure the absorbed and reflected sound energy in the external ear canal across a wide range of frequencies. WAI utilizes wideband clicks over a frequency range of 226-8,000 Hz as the probe. The functional status of the middle ear is analyzed by obtaining the wideband absorbance or energy reflectance. When sound waves are transmitted to the external ear canal, part of the acoustic energy is absorbed by the middle ear and cochlea, and the rest of the acoustic energy is reflected back to the external ear canal. The ratio between the reflected and total energy is defined as the energy reflectance (ER), where (1-ER) is defined as the wideband absorbance (WBA), which has values ranging from 1.0 to 0.0, representing total absorption of energy by the middle ear to total reflection [10]. Compared with the traditional tympanogram, WAI is more sensitive in evaluating different middle ear conductive function disorders [11–14]. Large numbers of studies on the application of WAI in newborns and infants have indicated that ER and WBA are more efficient in providing an accurate diagnosis of otitis media than are traditional single high-frequency immittance tests [15–18]. Beers et al. [19] compared the ERs of four different middle ear conditions, namely, normal, mild negative middle ear pressure, severe negative middle ear pressure, and middle ear effusion. The results indicated that there were significant differences between the ER under these middle ear conditions, suggesting that WAI is sensitive in the diagnosis of different levels of otitis conditions.

Recently, some scholars studied the characteristics of WAI in a population with conductive hearing loss but a normal eardrum and aerated middle ear cavity (e.g., otosclerosis, ossicular disarticulation, and superior canal dehiscence). Feeney et al. [11] first found that the WAI measurement results of otosclerotic patients were different from those of normal individuals, where ERs were increased at low frequencies (less than 1,000 Hz). Subsequently, Allen et al. [20] Shahnaz et al. [21] and Nakajima et al. [22] also reported similar findings [20–22], while Keefe et al. (2017) reported reduced ambient absorbance at 4 kHz in the otosclerotic group when compared with normal hearing controls [23]. Additionally, the study by Nakajima et al. [22] and Merchant et al. [24] suggested that the ER in the WAI test plays a critical role in differentiating the pathological causes of conductive hearing losses in those with normal ear drums (e.g., stapes footplate fixation, malleus fixation, ossicular disarticulation, and superior canal dehiscence). However, clinical data on WAI related to otosclerosis remains limited.

The ER and WBA in WAI are affected by race [19, 25, 26]. Although many studies on WAI have been conducted, these studies were mostly performed in the Caucasian population. Due to the influences of race, these results cannot be directly applied to the Chinese population. There are already some study results indicating significant differences in the WAI characteristics between Chinese individuals and Caucasians. For example, Beers et al. [19] compared the WAI data from Caucasian and Chinese children and found that the ERs of mid-range frequencies were significantly lower in Chinese children than in Caucasian children. Shahnaz and Bork [25] reported that the low-frequency ERs were higher in Chinese young adults than in Caucasian young adults, while the high-frequency ERs were significantly lower. These differences in ER could be attributed to the differences in body size between Caucasians and Chinese individuals [25]. Studies on body sizes using animal models indicated that animal body size is correlated with external ear canal volume, middle ear volume, eardrum area, and stapes footplate area, and an increase in physical volume is accompanied by an increase in the compliance of the aerated middle ear cavity [26, 27]. Therefore, the corresponding mechanoacoustical characteristics of the middle ear are changed.

To date, there have been no reports on WAI characteristics in Chinese individuals with otosclerosis. In the Chinese population, there are only WAI data on otitis media newborns and infants [28–32] or children and adults [19, 25], which have suggested racial differences. Whether the racial difference is associated with ER differences in WAI characteristics in Chinese otosclerotic patients compared with Western patients remains unclear. The purpose of the present study was to compare the ERs of patients with surgically confirmed otosclerosis with those of adults with normal middle ear function in the Chinese population to explore the effectiveness of WAI in the diagnosis of otosclerosis. Another aim of the present study was to compare our results with reported results obtained in Caucasians to provide further assistance in the clinical diagnosis of otosclerosis.

## 2. Materials and Methods

### 2.1. Subjects

Thirteen otosclerotic patients (20 ears) visiting the Department of Otorhinolaryngology, Peking Union Medical College Hospital, from May 2018 to November 2018 satisfied the inclusion criteria and were enrolled in the study. The inclusion criteria for the otosclerotic group were as follows: ① normal otoscopy exam results; ②conductive or mixed hearing loss according to PTA; ③negative Gelle's tests; ④type A (the static acoustic admittance: 0.03~1.6 mmho; peak pressure: -100~+100 daPa), As (the static acoustic admittance: <0.3 mmho; peak pressure: -100~+100 daPa), or Ad (the static acoustic admittance:>1.6 mmho; peak pressure: -100~+100 daPa) 226-Hz tympanograms [33]; ⑤no occupying, inflammatory or malformation lesions on CT exam; and ⑥stapes fixation observed in the following surgery. Among these individuals, there were 11 females (17 ears) and 2 males (3 ears). The ages of the otosclerotic patients ranged from 18 to 52 y, with an average age of 31.7±10.4 y. WAI assessments were performed before surgery. Thirteen individuals (26 ears) with normal middle ear function who were matched with respect to age and gender to those in the otosclerotic group were recruited as the control group. The age of the control group patients also ranged from 21 to 52 y, with an average age of 31.1±10.3 y. The inclusion criteria for the control group were as follows: ①air conduction threshold ≤25 dB HL at the frequency range from 250 to 8000Hz in PTA and an air-bone gap ≤10dB at the frequency range from 250 to 4000Hz in PTA; ②type A tympanograms and detection of an ipsilateral acoustic reflex; ③normal distortion-product otoacoustic emission results (signal-to-noise ratio >6dB in more than 4 out of 8 frequencies); and ④no history of otitis media or vertigo. Subjects in the control group also received WAI examinations. The use of human subjects in the present study was reviewed and approved by the institutional review board of Peking Union Medical College Hospital.

### 2.2. Test Method

#### 2.2.1. Equipment

The test equipment includes a Titan hearing test platform for WAI test (Interacoustics A/S, Middelfart, Denmark), a Madsen Conera audiometer (GN Otombtrics A/S, Taastrup, Denmark), and a Madsen Otoflex 100 Tympanometer (GN Otombtrics A/S).

#### 2.2.2. Test Methods

Criteria screening test: a Madsen Conera audiometer was employed for PTA and Gelle's tests. A Madsen Otoflex 100 Tympanometer was utilized for the 226-Hz tympanometry. PTA was performed in a sound-proof booth, where the background noise was less than 30dB(A). Tympanometry was performed in a quiet room where the background noise was less than 45dB(A). The equipment was calibrated yearly according to the instructions.

WAI test: a Titan hearing test platform produced by Interacoustics was employed to record WAI. The platform included a Titan unit, clinical probe extensions, integrated probes, and 0.2/0.5/2/5 cc couplers for calibration. Prior to the test, the probe was placed into each of the four couplers on the Titan module for calibration [14, 34]. The initial pressure was +200 daPa, and the final pressure was -600 daPa. The pressure change rate was 50 daPa/s from positive to negative. Ear plugs of appropriate size were used to seal the external ear canal before the Titan unit was started. The probe emitted wideband clicks with an intensity of 96 dB peSPL (frequency range of 226-8,000Hz). The energy that returned to the external ear canal was collected by the microphone on the probe. Collected data were processed by a preamplifier and an analog-to-digital converter prior to being input into a computer for further processing. The ER values under different frequency probe tones and pressures were obtained.

### 2.3. Data Analysis

ERs under both ambient and peak pressure were collected for WAI analysis. The peak pressure refers to the pressure under which the maximum admittance was got in each tympanogram. For each condition, the ERs of 16 frequencies of one-third octaves were collected for statistical analysis. The 16 one-third octave or close to one-third octave frequencies were 257, 324, 408, 500, 630, 794, 1,000, 1,260, 1,587, 2,000, 2,520, 3,175, 4,000, 5,040, 6,350, and 8,000 Hz.

SPSS17.0 was employed for statistical analysis. When comparing the ER differences in WAI between the otosclerotic and control groups, a mixed-model analysis of variance (ANOVA) and independent-samples *t*-test was used. Bonferroni correction was used for multiple comparisons. An independent-samples *t*-test was also used to compare age, 226-Hz static acoustic admittance and resonance frequency. Fisher's exact test was used to compare gender differences between the otosclerotic and control groups.

## 3. Results

### 3.1. Demographic and Clinical Audiology Data for Otosclerotic Subjects

There were no significant differences in age (*t*=0.131,* p*=0.897) and gender (*p*=1.000) between the otosclerotic and control groups. PTA and 226-Hz tympanometry results for all the otosclerotic ears enrolled in the present study are provided in Table 1. The 226-Hz tympanometry results indicated that 10(50%), 9(45%) and 1 (5%) of the ears yielded type As, A, and Ad tympanograms, respectively. The static acoustic admittance in the otosclerotic and control groups was 0.50±0.46 and 0.65±0.34 mmho, respectively. The independent-samples* t* test revealed no significant difference in the 226-Hz static acoustic admittance between the otosclerotic and control groups (*t*=1.249,* p*=0.218).

### 3.2. Comparing Wideband Energy Reflectance between Subjects with Normal Middle Ear Functions and Subjects with Otosclerosis

We used a mixed-model ANOVA to analyze the ER data. In this model, gender (male and female) and group (normal group and otosclerotic group) were between-subject factors, and pressure (ambient vs. peak) and frequency (16 frequencies of one-third octaves) were within-subject factors. The main effects of pressure (F=18.832,* p*≤0.001) and frequency (F=80.470,* p*≤0.001) were significant. However, the effects of gender (F=3.969,* p*=0.053) and group (F=0.700,* p*=0.407) were not significant. The interaction effects between group and frequency (F=7.861,* p*=0.007), frequency and pressure (F=10.383,* p*=0.002), and pressure and gender (F=4.691,* p*=0.036) were significant.

**Figure 1:**
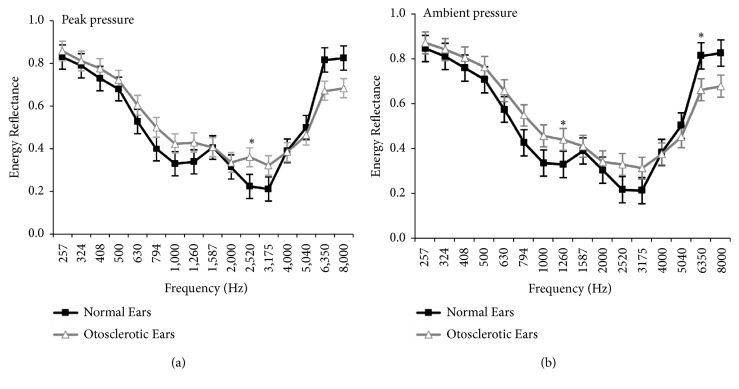
The mean and standard error of energy reflectance (ER) from 226 to 8000Hz are shown for both the otosclerotic and control groups. Panels (a) and (b) show the ERs when the external ear canal was under peak and ambient pressure, respectively. *∗*,* p* < 0.05/16 = 0.003,* Bonferroni corrected.*

To investigate the frequencies at which the group differences occurred, an independent-samples* t* test was performed. Bonferroni correction was used for multiple comparisons. When the external ear canal was under peak pressure, the ERs of frequencies less than 4,000 Hz in otosclerotic patients were higher than those in the control group. The ERs of frequencies above 4000 Hz in the otosclerotic group were less than those in the control group. Within all frequencies analyzed, the differences observed at 2,520 Hz was of statistical significance (*t*=-3.736,* p*=0.001,* p<0.05/16=0.003, Bonferroni corrected*) (Figure 1(a)). When the external ear canal was under ambient pressure, the ERs of all the analyzed frequencies had similar trends to those observed under peak pressure (Figure 1(b)). For the frequencies of 1,260 Hz (*t*=-3.390,* p*=0.002) and 6,350 Hz (*t*=3.166,* p*=0.003), the differences in ERs between the otosclerotic and control groups were statistically significant (*p<0.05/16=0.003, Bonferroni corrected*).

**Figure 2:**
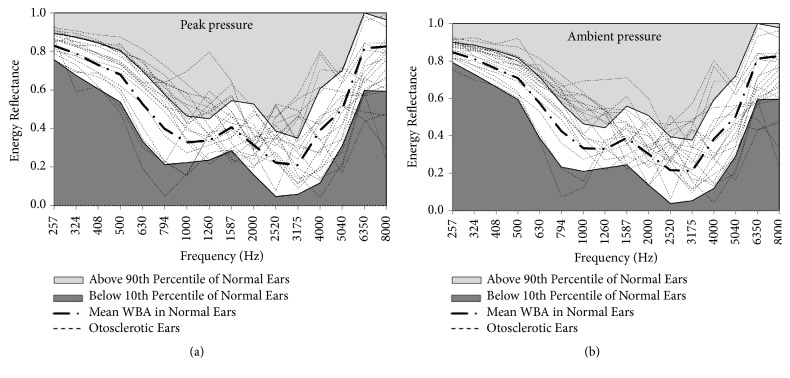
The individual energy reflectance for all 20 otosclerotic ears from 226 to 8000Hz. The mean energy reflectance and 80% range (10th to 90th percentile) for normal ears are also shown in this figure. Panels (a) and (b) show the ERs when the external ear canal was under peak and ambient pressure, respectively.

The 80% normal range (between the 10th and 90th percentiles) of ERs for the control group is illustrated in Figure 2. Other than otosclerotic ear numbers 15 and 17, all the otosclerotic ears had at least 1 frequency that fell outside of the 80% normal range.

### 3.3. Comparison of WAI Data Characteristics between Chinese Individuals and Caucasians with Normal Middle Ear Functions

**Figure 3:**
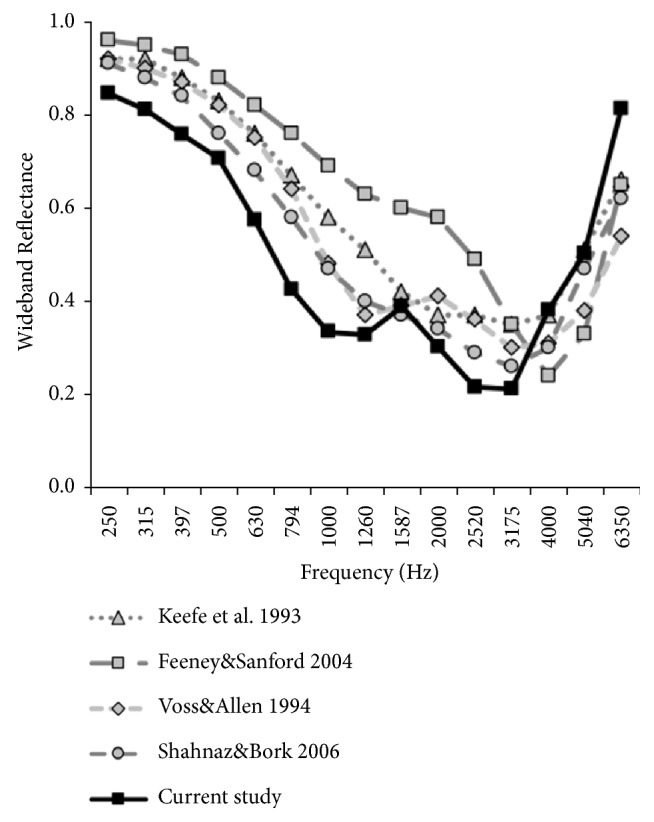
Comparison of mean energy reflectance in normal ears as a function of frequency between the current study and previous studies, including those by Shahnaz and Bork [25], Keefe et al. [35], Voss and Allen [10], and Feeney and Sanford [36].

The ERs collected from Chinese subjects with normal hearing were compared with the WAI data reported by Shahnaz and Bork [25], Keefe et al. [35], Voss and Allen [10], and Feeney and Sanford [36] (Figure 3). As illustrated in Figure 3, for frequencies less than 4,000 Hz, the ERs of Chinese patients measured in our study were apparently lower than those of Caucasian subjects. For frequencies above 4,000 Hz, the overall trend of ERs measured in our study was higher than the results collected in studies on Caucasians.

### 3.4. Comparison of Resonance Frequencies between Subjects with Normal Middle Ear Function and Patients with Otosclerosis

The average resonance frequency in the otosclerotic group was 961±240Hz, whereas that in the control group was 854±152Hz. The independent-samples* t* test revealed no significant difference in the resonance frequencies between the otosclerotic and control groups (*t*=-1.840,* p*=0.073).

## 4. Discussion

In the present study, the characteristics of WAI in Chinese otosclerotic patients were studied. The results indicated that the ERs of certain low frequencies in Chinese otosclerotic patients were significantly higher than those in the control group. The alterations displayed in the Chinese otosclerotic patients were similar to those reported in studies on Caucasians, but the norms collected in the present study were different from those in previous studies. There were no significant differences observed in resonance frequencies between otosclerotic patients and the control group. Specific analyses are discussed below.

In our study, we analyzed the traditional 226-Hz tympanograms obtained from otosclerotic patients. A total of 45% (9/20) of the patients yielded normal admittance (type A), 50% (10/20) showed reduced admittance (type As), and 5% (1/20) showed elevated admittance (type Ad). Such findings suggested the presence of variations in middle ear dynamic characteristics in otosclerotic patients. Additionally, almost half of the otosclerotic patients yielded normal admittance, which resulted in a large degree of overlap with subjects having normal middle ear functions. These data further confirmed the limitations of applying traditional 226-Hz tympanometry in the diagnosis of otosclerosis, which is consistent with results from previous studies [3, 4].

For WAI, wideband clicks in the frequency range of 226-8,000 Hz were employed as the probe, which allows WAI to provide more information than traditional single-frequency probe tones, due to the wider range of probe frequencies. The status of middle ears was analyzed by obtaining WBAs or ERs. The results of the present study showed that the ERs of frequencies 1,260 and 2,520Hz were significantly higher in otosclerotic patients than those in the control group, whereas the ERs of 6,350 Hz was significantly lower in otosclerotic patients. These findings suggested that WAI could provide useful information in distinguishing otosclerotic and normal ears. Based on the results provided in Figure 2, all but 2 otosclerotic ears had at least 1 frequency that fell outside of the 80% normal range. These results further suggest the presence of unique characteristics in WAI results obtained from otosclerotic patients compared with individuals with normal middle ear functions. However, the number of cases included in our study was still insufficient for evaluating sensitivity and specificity; thus, further studies are still necessary.

The above-mentioned results found in our study were not quite consistent with those obtained in studies on Caucasians. The results of former research on the application of WAI to Caucasian otosclerosis patients indicated that the ERs of low frequencies (less than 1,000 Hz) were significantly higher in otosclerosis patients than in normal individuals [11, 20, 21]. It was speculated that the increase in ER in otosclerotic patients may be due to the increase in the stiffness of the annular ligament. The increased stiffness leads to a greater degree of acoustic impedance mismatch and increased ER to the external auditory canal for frequencies less than 2 kHz [20]. While another study revealed WBA at 4 KHz was significantly lower in otosclerotic patients than normal group, which meant ER at 4KHz was significantly higher in otosclerotic patients than normal group. The reduced WBA at 4 KHz may be due to functional differences in ossicular-chain transmission at frequencies above the dominant resonance of the TM [23]. In our study, the ERs of frequencies less than 4,000 Hz were also higher in otosclerosis patients than in normal individuals, however, the differences were only significant at frequencies of 1,260 and 2,520Hz. In addition to the similar trends at the low-mid frequencies, the ERs were observed to be significantly lower in otosclerotic patients than those in normal individuals at high frequency (6,350 Hz), which was rarely reported in previous studies. Feeney et al.'s [11] study observed higher ER in otosclerotic patients in high frequencies. However, their observation was inconsistent with our findings. Previous studies seldom recommended using the ERs at high frequencies as diagnostic criteria due to their high variability [20]. Thus, further study is still needed to verify the effectiveness of high frequencies in the diagnosis of otosclerosis.

We compared data obtained from the control group in our study with those reported in the literature [10, 25, 35, 36]. Comparison of the results indicated that for frequencies less than 4,000 Hz, the ER in Chinese individuals with normal middle ear functions was less than that in Caucasian individuals. For frequencies greater than 4,000 Hz, the ERs measured in our study were higher than those measured in Caucasians in other studies (Figure 3). The norms in our study were different from previous studies [10, 25, 35, 36]. The major cause of this phenomenon is probably the racial difference of subjects. We included a Chinese population, while Caucasian individuals were the subjects in previous studies. According to previous studies, the differences were attributed to the body size differences between the races [25]. Chinese individuals are typically physically smaller than Caucasians, and a smaller body size leads to less compliance of the middle ear cavity. The corresponding mechanoacoustical characteristics of the middle ear are then changed [25, 27, 37, 38]. Although differences in WAI data were observed between Caucasians and Chinese individuals, the results obtained in our study were different from those collected by other scholars. The results from the study by Beers and Shahnaz indicated that the ERs of mid-frequencies in Chinese children with normal hearing were significantly lower than those in Caucasian children [19]. Shahnaz and Bork [25] reported that the low-frequency ERs in Chinese young adults were significantly higher than those in Caucasian young adults, while their high-frequency ERs were significantly lower than those in Caucasian young adults. These differences could be attributed to the following factors. First, the age and gender of the subjects were different. In our study, subjects with normal hearing who were gender- and age-matched with otosclerotic patients were included. Among the included subjects with normal hearing, there were 11 females (22 ears) and 2 males (4 ears), ranging in age from 21-52 y (average: 31.1±10.3y). In the study by Beer et al. [19] the subjects were children, ranging in age from 5 y and 1 month to 6 y and 11 months (average: 6.15 y). In Shahnaz et al.'s [26] study, they included young adult Caucasians and Chinese individuals aged 18-32 y. A previous study indicated that there were differences in the ER between young adults and seniors [36]. The ER in the senior group was less than that in the young adult group at 800-2,000 Hz yet slightly higher at approximately 4,000 Hz. This result indicated the reduction in the stiffness of the middle ear system with age [36]. In addition, different instruments may lead to different results. In our study, the Interacoustics system was employed to record WAI, while the Mimosa Acoustics system was employed in previous studies [19, 25]. Some studies reported that Caucasian subjects measured using the Interacoustics device differed significantly from those measured using the Mimosa device at 5000 Hz. Chinese subjects measured using each device did not differ significantly at any frequency [25]. Thus, although both studies compared WAIs between Caucasians and Chinese individuals, the results could be different.

Currently, there is no common agreement regarding the conditions for ER measurements, e.g., under ambient or peak pressure [21, 26]. In our study, the pressure had a significant effect on the ERs. Under peak pressure, 1 ER frequencies showed significant differences between the otosclerotic and control groups, while under ambient pressure, 2 ER frequencies showed significant differences between the two groups. This result implied that ER differences under ambient pressure were more obvious than those under peak pressure. However, whether ERs under ambient pressure were better than those under peak pressure still needs further investigation due to the limitation of the sample size in the present study.

Analyses of the middle ear resonance frequency in otosclerotic patients were also performed in our study, and the resonance frequencies in otosclerotic patients were slightly higher than those in normal individuals but without statistical significance. Regarding resonance frequency, previous studies showed various results. Shahnaz and Polka [3] reported significantly higher resonances in otosclerotic patients. However, there are also studies that reported no significant difference, or even lower values, in the resonance frequencies of otosclerotic patients versus the control group [1]. These discrepancies could be attributed to the pathological stages of selected otosclerotic cases [39]. As mentioned above, different stages of pathological changes in otosclerosis could correlate with various middle ear dynamic characteristics, including high, normal, and even low stiffness [1]. The results in our study indicated that although the pathological changes in otosclerosis could lead to changes in resonance frequency, there was a large overlap between the resonance frequency ranges in otosclerotic patients and normal individuals. Therefore, changes in resonance frequency cannot serve as an effective and independent tool for the diagnosis of otosclerosis.

## 5. Conclusions

In the present study, significant differences in ERs were detected in Chinese otosclerotic patients compared with normal hearing Chinese individuals, indicating that WAI could provide useful information for the diagnosis of otosclerosis in the Chinese population. Although the trends in the ER data in the Chinese patients were similar to those observed in the Caucasian patients, the norms found in the present study were different from those in the Caucasians using the Mimosa Acoustics system. These results suggested that the norms and diagnostic criteria corresponding to the patient's racial group and WAI testing system should be referenced when evaluating middle ear function to improve the efficiency of WAI in the diagnosis of otosclerosis.

## Figures and Tables

**Table 1:** The demographic and audiologic data in patients with otosclerosis.

Number	L/R	gender	Age (year)	The type of Tympanogram (226Hz)	Threshold of pure tone audiometry (dB HL)					
					250 Hz	500 Hz	1000 Hz	2000 Hz	4000 Hz	8000 Hz
					AC BC	AC BC	AC BC	AC BC	AC BC	AC
1	L	F	51.9	A	60 10	60 15	55 25	40 35	45 20	50
2	R	F	51.9	Ad	55 10	55 15	50 20	40 30	35 15	50
3	L	M	31.4	A	50 0	50 5	50 10	45 25	60 45	80
4	L	F	25.6	As	50 0	55 0	55 5	45 20	30 5	20
5	R	F	25.6	As	50 0	55 5	50 5	40 20	15 5	15
6	L	F	35.8	A	60 10	55 15	55 20	60 30	50 25	80
7	R	F	35.8	A	65 20	60 25	60 30	50 30	55 30	65
8	R	F	33.2	As	65 10	60 10	50 10	50 20	45 15	40
9	L	F	29.3	A	60 0	60 0	50 5	45 20	40 10	35
10	R	F	29.3	As	60 0	60 0	60 10	55 20	45 10	40
11	R	F	18.5	A	45 5	45 0	40 5	30 10	20 0	15
12	L	F	20.7	As	55 10	60 10	60 25	40 25	25 15	25
13	R	F	20.7	As	60 10	60 15	65 25	40 25	25 10	20
14	L	F	41.7	A	60 15	65 10	65 15	55 25	35 15	45
15	R	F	41.7	As	60 10	60 15	65 20	55 20	45 15	45
16	L	F	23.1	As	60 10	55 20	55 30	50 30	40 25	55
17	L	F	21.3	As	55 10	60 15	50 20	35 30	25 10	40
18	L	F	47.6	As	75 20	80 25	85 40	70 45	60 30	75
19	L	M	32.3	A	55 5	60 10	65 20	50 35	60 40	70
20	R	M	32.3	A	55 5	65 10	65 25	50 35	45 30	45

L=left ear; R=right ear; AC=air conduction; BC= bone conduction; F=female; M=male.

## Data Availability

The data used to support the findings of this study are available from the corresponding author upon request.
